# Oscillatory Bursts in Parietal Cortex Reflect Dynamic Attention between Multiple Objects and Ensembles

**DOI:** 10.1523/JNEUROSCI.0231-20.2020

**Published:** 2020-09-02

**Authors:** Andreas Wutz, Agnese Zazio, Nathan Weisz

**Affiliations:** ^1^Centre for Cognitive Neuroscience, University of Salzburg, Salzburg, 5020, Austria; ^2^Picower Institute for Learning & Memory, Massachusetts Institute of Technology, Cambridge, Massachusetts 02139; ^3^Cognitive Neuroscience Section, Istituto Di Ricovero e Cura a Carattere Scientifico, Istituto Centro San Giovanni di Dio Fatebenefratelli, Brescia, 25125, Italy

**Keywords:** dynamic attention, ensemble attention, multiple-object attention, neural oscillations, parietal cortex

## Abstract

The visual system uses two complimentary strategies to process multiple objects simultaneously within a scene and update their spatial positions in real time. It either uses selective attention to individuate a complex, dynamic scene into a few focal objects (i.e., object individuation), or it represents multiple objects as an ensemble by distributing attention more globally across the scene (i.e., ensemble grouping). Neural oscillations may be a key signature for focal object individuation versus distributed ensemble grouping, because they are thought to regulate neural excitability over visual areas through inhibitory control mechanisms. We recorded whole-head MEG data during a multiple-object tracking paradigm, in which human participants (13 female, 11 male) switched between different instructions for object individuation and ensemble grouping on different trials. The stimuli, responses, and the demand to keep track of multiple spatial locations over time were held constant between the two conditions. We observed increased α-band power (9-13 Hz) packed into oscillatory bursts in bilateral inferior parietal cortex during multiple-object processing. Single-trial analysis revealed greater burst occurrences on object individuation versus ensemble grouping trials. By contrast, we found no differences using standard analyses on across-trials averaged α-band power. Moreover, the bursting effects occurred only below/at, but not above, the typical capacity limits for multiple-object processing (at ∼4 objects). Our findings reveal the real-time neural correlates underlying the dynamic processing of multiple-object scenarios, which are modulated by grouping strategies and capacity. They support a rhythmic, α-pulsed organization of dynamic attention to multiple objects and ensembles.

**SIGNIFICANCE STATEMENT** Dynamic multiple-object scenarios are an important problem in real-world and computer vision. They require keeping track of multiple objects as they move through space and time. Such problems can be solved in two ways: One can individuate a scene object by object, or alternatively group objects into ensembles. We observed greater occurrences of α-oscillatory burst events in parietal cortex for processing objects versus ensembles and below/at versus above processing capacity. These results demonstrate a unique top-down mechanism by which the brain dynamically adjusts its computational level between objects and ensembles. They help to explain how the brain copes with its capacity limitations in real-time environments and may lead the way to technological innovations for time-critical video analysis in computer vision.

## Introduction

**Figure 1.**
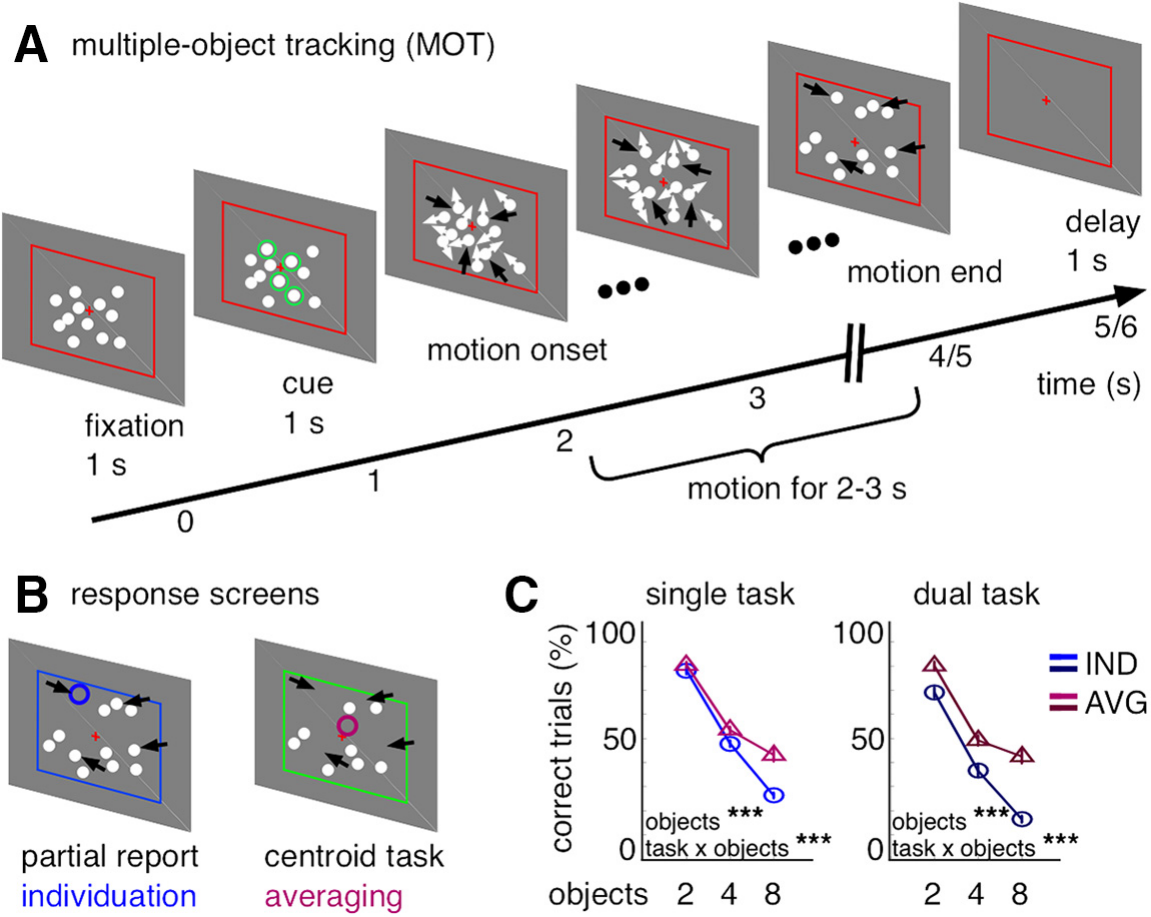
Trial sequence, experimental conditions, and behavioral data. ***A***, A typical trial in the experiment. Black arrows indicate the target-object locations and are only shown for illustration. ***B***, Response screens for each task. Blue and red circles represent the probe locations: the location of the item to be indicated for partial report (blue) for the individuation condition or the centroid location of the items (red) for the averaging condition. They are only shown for illustration. ***C***, Behavioral performance (in percent correct trials) per task and set size under single-task (left) and dual-task conditions (right). Error bars indicate 1 SE for repeated measures (Morey, 2008). ***p < 0.001.

Dynamic perceptual experiences require the visual system to process multiple objects simultaneously within a scene and update them from moment to moment. Such situations provide a high degree of ecological validity for real-world visual cognition because we often encounter them in everyday life, for example, in traffic situations or in team sports (Scholl, 2009). Moreover, dynamic multiple-object scenarios are an important problem in computer vision with wide applications in the time-critical video analysis for robotics or autonomous driving (Kazanovich and Borisyuk, 2006; Xiang et al., 2015). An ideal task to investigate this ability in experimental settings is multiple-object tracking (MOT) (Pylyshyn and Storm, 1988). In a typical MOT trial, participants need to keep track of multiple, randomly moving target-objects among distractors for a period of several seconds (see Fig. 1*A*).

Previous research has identified two distinct key strategies for the visual system to deal with dynamic multiple-object problems. A first important mechanism for MOT is object individuation, which involves selecting features from a crowded scene, binding them into an object, and individuating it from other objects (for review, see Xu and Chun, 2009; Wutz and Melcher, 2014). Individuation provides local detail about specific objects in a scene, but only a few objects (typically ∼4) can be individuated at once (Cowan, 2010) because of limited selective attention (Cavanagh and Alvarez, 2005). Indeed, object-based attention and individuation are a central component to visual cognition, since its capacity limitations set the bounds for many visual tasks, for example, enumeration (Jevons, 1871), visual working memory (Luck and Vogel, 1997), and MOT (Trick and Pylyshyn, 1994; for review, see Piazza et al., 2011). For MOT, individuation capacity is often probed by indicating the spatial location of one object location from the pool of target-objects at trial end (i.e., partial report; see Fig. 1*B*).

A second core mechanism for MOT is to represent multiple objects as an ensemble or group (Yantis, 1992; Merkel et al., 2014, 2015, 2017). In contrast to focal attention to individual objects, recent reports have highlighted the ability for information integration and compression in the visual scene analysis by computing group-level ensemble statistics across multiple objects (for review, see Alvarez, 2011; Whitney and Yamanashi Leib, 2018). For example, the average size (Ariely, 2001; Chong and Treisman, 2003), orientation (Parkes et al., 2001), and location (Alvarez and Oliva, 2008) of many objects can be reported with high precision and velocity, and often better compared with the properties of individual objects. Critically, ensemble computations exploit higher-order regularities diagnostic of the large-scale scene-layout (its “gist”) (Torralba and Oliva, 2003; Greene and Oliva, 2009), which can be computed even under conditions of reduced or withdrawn attention to individual, focal objects and thus overcome its typical capacity limitations (Alvarez and Oliva, 2009; Corbett and Oriet, 2011). A typical ensemble-statistic for MOT is the average location of all target-objects (i.e., their centroid; see Fig. 1*B*).

Currently, there is no clear consensus about the neural correlates underlying dynamic multiple-object problems (e.g., MOT) and its processing differences in terms of focal object-level versus distributed ensemble-level attention. Likewise, it is not clear how its different processing capacity limitations are implemented in neural function and whether they are because of “online” perceptual or “offline” memory-related mechanisms. Previous fMRI and MEG work on multiple-object attention on the level of object individuation suggests neural substrates in parietal cortex (Todd and Marois, 2004; Xu and Chun, 2009; Rouhinen et al., 2013). In terms of temporal dynamics, neural oscillations in the α-frequency band (9-13 Hz) are a key candidate because they are associated with the functional inhibition of neural processes (Klimesch et al., 2007; Haegens et al., 2011). In the case of dynamic object individuation, neural excitation-inhibition needs to be regulated focally, to carve-out discrete objects from continuous visual inputs (e.g., by modulating signal-to-noise in saliency maps) (Quiles et al., 2011; Jensen et al., 2012; Wutz et al., 2014) and keep distinct objects separate in spatial working memory (Palva and Palva, 2007; Jensen et al., 2014). By contrast, ensemble computations require more global inhibition, because attention is distributed across the visual scene and objects are represented as a group (Torralba and Oliva, 2003; Alvarez, 2011).

We aimed to identify the neural dynamics underlying object- and ensemble-level computations during MOT by means of MEG recordings. We focused on single-trial activity changes in neural oscillations (e.g., oscillatory burst events) (Lundqvist et al., 2016; for review, see van Ede et al., 2018), because they account for relevant trial-by-trial variability in neural timing expected in time-critical tasks, such as MOT. The participants switched between instructions to perform object individuation(i.e., partial report) or ensemble processing (i.e., centroid/averaging task; see Fig. 1*A*,*B*) in counterbalanced blocks and in a dual-task condition, in which the task demands were randomly intermixed in each block and cued after each trial, to control for top-down influences. This strategy allowed us to match processing in the two tasks in visual stimuli, responses, and the requirement to keep track of multiple spatial locations over time, while preserving critical differences in the dynamic deployment of attention to individual objects versus group-level ensembles during dynamic multiple-object problems.

## Materials and Methods

All procedures were approved by the ethics committee of the University of Salzburg.

### 

#### Participants

Twenty-four participants (13 female; mean age ± SD, 25.9 ± 5.3 years; 20 right-handed) took part in the experiment. All participants had normal or corrected-to-normal vision, gave written informed consent before the experiment, and received a monetary compensation or course credits.

#### Apparatus

##### MEG data acquisition

Electrophysiological activity was recorded with a whole-head MEG system with 102 magnetometers and 204 planar gradiometers (Neuromag306; Elekta), sampled at 1000 Hz in a magnetically shielded room. For each participant, a head frame coordinate reference was defined before the experiment by digitizing the cardinal points of the head (nasion and left and right preauricular points), the location of five head position indicator coils, and a minimum of 200 other head shape samples (3Space Fastrack; Polhemus). The head position within the MEG helmet (relative to the head position indicator coils and the MEG sensors) was controlled before each block to ensure that no large movements occurred during the data acquisition.

##### Stimulus presentation

Stimuli were generated using MATLAB 8.5 (The MathWorks) and Psychophysics Toolbox, version 3 (Brainard, 1997; Pelli, 1997). A DLP projector (PROPixx; VPixx Technologies) showed the stimuli at a refresh rate of 120 Hz centered onto a translucent screen (25 horizontal × 16 vertical degrees of visual angle [DVA]). The screen was located in front of the participant (viewing distance, 125 cm) within the dimly lit, magnetically shielded MEG room. Stimulus timing was controlled with a data and video processing peripheral (DATAPixx; VPixx Technologies) and monitored via a photograph diode placed at the upper left corner of the projection screen. The delay between trigger and stimulation onset was corrected with this method.

#### Stimuli and experimental design

Each trial started with the central presentation of a red fixation cross (0.25 visual angle, DVA) on a gray background, of a red frame indicating the bounds of the tracking display (6 × 6 DVA) and of 12 white dots (0.25 DVA) presented at random, nonoverlapping locations within the inner 3 × 3 DVA of the display for 1 s duration. Then, a subset of the white dots (2, 4, or 8 depending on the target-object pool per trial) was cued as task-relevant objects by presenting a green circle (0.5 DVA) around them for 1 s duration. The total number of presented items was held constant, to control for visual load and track set size effects based exclusively on the number of task-relevant items. Subsequently, the cue was removed, rendering task-relevant and -irrelevant objects indistinguishable from each other, and the dots started to move with 1 DVA/s speed in random directions on linear paths. The motion paths were generated offline before the experiment such that the moving dots did not collide with each other (0.5 DVA minimal center-to-center distance) or with the display edges. The duration of the motion epoch was randomly jittered to last between 2 and 3 s duration per trial such that the objects' final spatial positions were unpredictable during tracking. It was followed by a delay epoch (for 1 s duration), during which only the fixation cross and the tracking boundary were presented and the subjects had to remember the target dot locations (see Fig. 1*A*). At trial end, a response screen was presented, which depended on the performed task (i.e., object individuation, ensemble processing). On individuation trials, all distractor dots and all except for one, *a priori* unknown target dot, were presented after the delay, and the subjects' task was to indicate the location of the missing target dot from the pool of target-objects via mouse-click (i.e., partial report). On ensemble averaging trials, all distractor dots were reshown and the subject's task was to indicate the centroid location of the target dots (see Fig. 1*B*). The two tasks were run in counterbalanced blocks and in a dual-task condition, which controls for strategic influences, because the task demands were randomly intermixed within a block and cued after each trial. We used two blocks each comprising 120 trials for each condition (240 trials total for the two single tasks and the dual-task) with all set sizes (2, 4, 8 objects) balanced and randomly intermixed within a block. The experiment lasted ∼2.5 h.

#### Behavioral data analysis

We quantified behavioral performance in terms of percent correct trials, in which the difference between the responded and the probed screen location (i.e., partial report item or centroid) was <0.5 DVA. Performance was contrasted between the tasks and across the different object-pools with a two-way, repeated-measures ANOVA. The performance difference per task between single- versus dual-task conditions was tested with dependent-samples *t* test. All error bars (for both behavioral and MEG measures) show the SEM for repeated measures. The mean between conditions was subtracted from the data in each condition before calculating the SE. The resulting error estimate was bias corrected by the number of conditions [M, multiplied by √(M/(M − 1))] (Morey, 2008).

#### Eye-movement data analysis

Eye-movements were recorded with a 2 kHz, binocular eye-tracker (TRACKPixx; VPixx Technologies). We used an automated algorithm for micro-saccade detection that computes thresholds based on velocity changes from the horizontal and vertical eye-tracker components (Engbert and Kliegl, 2003). Micro-saccades were identified as binocular events that exceeded a given duration and velocity threshold. The minimum micro-saccade duration was 12 ms, and the velocity threshold was set at 6 times the noise level per trial and horizontal/vertical component. Trials that contained blinks were excluded from the analysis before micro-saccade detection (on average, 44 ± 36 SD trials were excluded). Because of technical difficulties, eye-tracking data were only available for 13 of 24 subjects. On average, the participants performed 209 micro-saccades over the course of the experiment (SD = 202, minimum = 12, maximum = 534). For each participant, we calculated the difference in the vertical and horizontal eye-gaze position before and after each micro-saccade during the motion and jitter epochs, to quantify the micro-saccade size per trial. Vertical and horizontal eye gaze components were combined via vector addition. Moreover, we averaged the simultaneously recorded α power over the time samples corresponding to micro-saccades per trial. Then, we correlated α power and micro-saccade size across all micro-saccade occurrences for each participant separately. The false discovery rate procedure by Benjamini and Hochberg (1995) was used to correct for multiple comparisons across participants.

#### MEG data analysis

##### Data preprocessing

The data were analyzed using custom-built MATLAB code (MATLAB 9.5; The MathWorks) and the FieldTrip toolbox (Oostenveld et al., 2011). We use a signal space separation algorithm implemented in the Maxfilter program (version 2.2.15) provided by the MEG manufacturer to remove external noise from the MEG signal (mainly 16.6 Hz, which is the alternating current used by the railway electrification systems in Austria, Germany, and Switzerland, and 50 Hz plus harmonics, which is line noise in Europe) and realign data to a common standard head position (trans default Maxfilter parameter) across different blocks based on the measured head position at the beginning of each block. Data were segmented from 1.5 s before to 6 s after fixation onset and downsampled offline to 250 Hz. In separate analysis, data were time-locked to the motion epoch offset/delay epoch onset and segmented from 3 s before to 3 s after, to reveal exclusive delay epoch activity. Data were high-pass filtered at 1 Hz with a FIRWS filter (2 Hz transition width) and band-stop filtered between 49-51, 99-101, and 149-151 Hz with a two-pass Butterworth filter (order 4), applied in the forward and reverse directions. The trial average was subtracted from each single trial, to obtain induced activity without the contribution from stimulus-evoked components. For validation purposes, we reran the main bursting analyses without subtracting the event-related field and obtained very similar results. Unless otherwise indicated, all data show MEG activity on gradiometer channels. The effects on magnetometer channels were qualitatively and quantitatively similar.

##### Artifact rejection

A set of different summary statistics (variance, maximum absolute amplitude, maximum *z* value) was used to detect statistical outliers of trials and channels in the datasets. These trials and channels were removed from each dataset (semiautomatic artifact rejection). In addition, the data were visually inspected, and any remaining trials and channels with artifacts were removed. On average, 1.3 channels (±1.5 SD) and 9.7% of the trials (±2.6% SD) were rejected. The rejected channels were interpolated with the nearest-neighbors approach for sensor-level analysis. Interpolated channels were not used for source modeling.

##### Time–frequency representations

Time–frequency representations were calculated on single-trial data using Morlet-Wavelets applied to short sliding time windows in steps of 10 ms in the time interval between −0.5 and 5 s relative to fixation onset and in the frequency range between 5 and 50 Hz. We used a frequency-dependent window width of 5 cycles per frequency. The squared absolute value gave the signal power for each MEG sensor across different frequencies and time points. For gradiometer sensors, the power values were calculated for the horizontal and vertical component of the planar gradient and then combined via their vector sum. Peaks in the power spectra during the motion epoch (2–4 s) for each participant were found with an automatic peak-detection algorithm (i.e., with the in-built MATLAB function, *findpeaks.m*).

##### Neural activity increase with Wilcoxon sign-rank test

**Figure 2.**
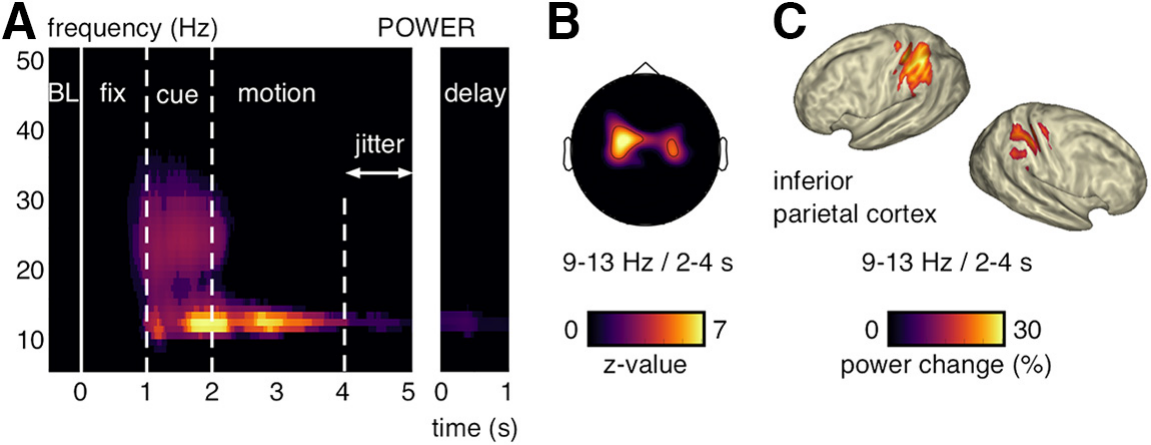
α-band power increase during MOT on the sensor and source level. ***A***, Power increase relative to pretrial baseline epochs (Wilcoxon *z* value) averaged over significant gradiometer sensors (see ***B***). ***B***, MEG-gradiometer topography for the power increase relative to pretrial baseline epochs (Wilcoxon *z* value) averaged between 9 and 13 Hz and between 2 and 4 s. ***A***, ***B***, *z* values with *p* < 0.05 are shown (Bonferroni-corrected). ***C***, Source modeling for the power increase relative to pretrial baseline epochs (signal change in percent) averaged between 9 and 13 Hz and between 2 and 4 s.

MEG signal power during the MOT task (i.e., fixation epoch [0–1 s], cue epoch [1–2 s], motion epoch [2–4 s], jittered motion-delay transition epoch [4–5 s], delay epoch [1 s duration time-locked to the end of the motion epoch on each trial]) was compared with the pretrial baseline epoch (−0.5 to 0 s relative to fixation onset) by means of a Wilcoxon signed-rank test. This time epoch was chosen as baseline because it was free from stimulus-evoked, eye-movement-related, and task-related activity. Baseline activity for each trial was calculated by averaging power between −0.5 and 0 s for every frequency bin on each sensor. Single-trial baseline values were then compared with each time–frequency bin and time sample during the task epochs. The sum of the signed rank difference across trials (Wilcoxon test statistic) was converted into a *z* value for a standard normal distribution. The resulting time–frequency *z* value maps for each sensor wereaveraged over subjects. Gradiometer and magnetometer sensor systems were analyzed separately. In order to identify sensors with significant activity increases, we averaged the *z* value maps over the α-frequency band (9-13 Hz) and the motion epoch (2–4 s). The resulting sensor topographies were Bonferroni-corrected for multiple comparison correction (threshold at *z* = 3.3 corresponding to one-sided *p* < 0.05/102 sensors; see Fig. 2*B*). Figure 2*A* shows the time–frequency *z* value map averaged over significant gradiometer sensors and masked at a Bonferroni-corrected threshold (*z* = 5.48 corresponding to one-sided *p* < 0.05/46 frequency bins × 500 time samples).

##### Neural activity increase with dependent-samples *t* test

The Wilcoxon sign-rank test quantifies the α power increase from baseline across trials (i.e., a fixed-effects analysis). For validation purposes, we also performed a random-effects analysis to quantify the α power increase (i.e., a dependent-samples *t* test over participants). For each participant, we computed the average power over all trials, the α-frequency band (9–13 Hz) and separately for the baseline (−0.5 to 0 s) and the motion epochs (2–4 s) per MEG-gradiometer channel. Then, we performed dependent-samples *t* tests (BL vs motion) across participants (df = 23). The two methods (fixed vs random effects) yield similar results with respect to the MEG-sensor selection.

##### Neural activity increase with bursting analysis

We followed the approach by Lundqvist et al. (2016) to identify oscillatory bursts in the MEG signal. Bursts were detected when the power per frequency bin and time sample during the MOT task epochs exceeded its respective pretrial mean power (averaged between −0.5 and 0 s) by 2× its trial-specific variability estimate for at least three cycles of the corresponding frequency. The variability estimate per trial and frequency were calculated by the SD of the MEG signal during the baseline epoch (−0.5 to 0 s; Eq. 1).

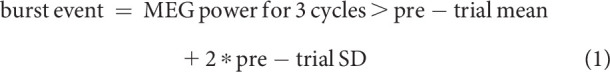


**Figure 3.**
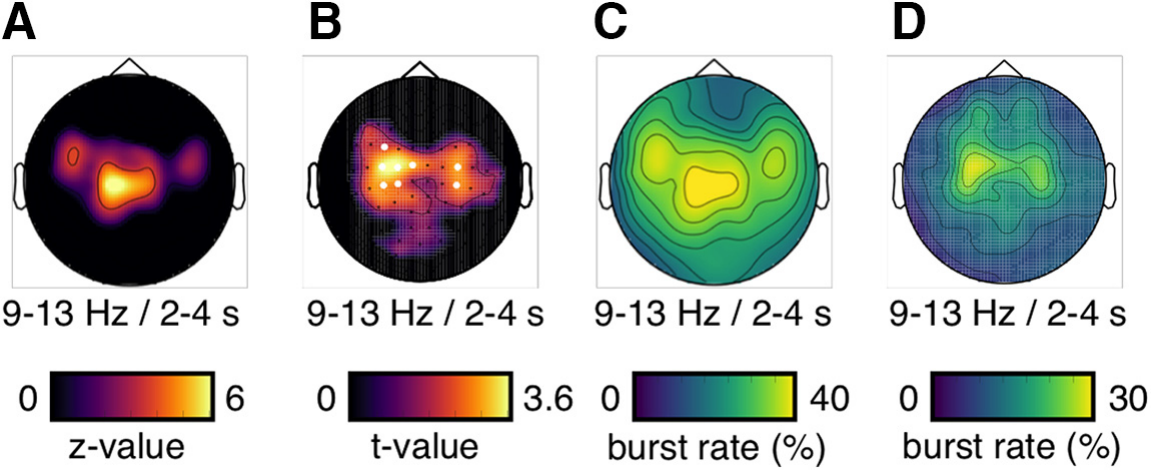
MEG-sensor topographies for power and bursting. ***A***, Magnetometer topographies for the power increase relative to pretrial baseline epochs (Wilcoxon *z* value) averaged between 9 and 13 Hz and between 2 and 4 s. ***B***, Gradiometer topographies for the power increase relative to pretrial baseline epochs with a random-effects approach (dependent-samples *t* test over participants) averaged between 9 and 13 Hz and between 2 and 4 s. For comparison, white dots indicate the sensors of interest resulting from the sensor selection with the fixed-effects approach (Wilcoxon test over trials; see Fig. 2*B*). ***C***, Magnetometer topographies for the burst rate (in percent trials) averaged between 9 and 13 Hz and between 2 and 4 s. ***D***, Gradiometer topography for the burst rates (in percent trials) without event-related field subtraction averaged between 9 and 13 Hz and between 2 and 4 s.

The bursting analysis results in sparse time–frequency maps for each trial and sensor, which separate the occurrence of a suprathreshold burst event from nonburst periods. In order to show the topographical distribution of burst events, we averaged the burst event maps over trials (i.e., calculating the burst rate), the α-frequency band (9–13 Hz), and the motion epoch (2–4 s). Figure 3*A* shows the time–frequency burst rate map averaged over gradiometer sensors with a significant power increase (Wilcoxon *z* statistic; see Fig. 2*B*) and masked above a burst rate of 25% trials. For validation purposes, we also used an alternative algorithm for burst event detection (derived from automatic spike-detection methods, adapted from Quiroga et al., 2004) (Eq. 2).

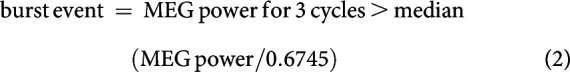


Equation 2 refers to the statistical concept of the probable error, which defines the half-range interval around a distribution's central point. For symmetrical distributions, it is equivalent to the interquartile range or the median absolute deviation (Hoaglin et al., 1983). The probable error can be expressed in terms of SDs when scaled with a constant factor that depends on the assumed distribution (i.e., this factor refers to the 75% quantile for a normal distribution with ϕ^−1^ (0.75) = 0.6745). It is a more robust measure of variability compared with the SD because it is more resilient to outliers (i.e., it remains largely constant across different burst rate regimens and burst amplitudes). Moreover, because the variability per trial and channel is estimated across the MEG signal of the entire trial, it is independent from the trial-specific baseline epoch. The two methods yielded highly similar results (compare Figs. 6 and 7).

##### Comparison between across-trials averaged power and single-trial bursting

As outlined above, the bursting analysis provides a single-trial measure of cortical activation. We compared the single-trial bursting findings to a standard approach on across-trials averaged power. For the power calculation, we first subtracted the average power in the baseline epoch (−0.5 to 0 s) from the power maps per condition. Thus, the power analysis accounts for possible average baseline differences between the conditions but, unlike the bursting analysis, it does not relate the single-trial MEG activity increases to its trial-specific baseline mean and variability estimate.

##### Source localization

To illustrate the source localization of the observed sensor-level effects, we performed source modeling based on a spatial-filtering algorithm (Beamforming). A structural MRI was available for 16 of 24 participants. We coregistered the brain surface from their individual segmented MRIs with a single-shell head model (Nolte, 2003). For the remaining subjects, we obtained the canonical cortical anatomy from the affine transformation of an MNI template brain (brainweb.bic.mni.mcgill.ca/brainweb/) to the subject's digitized head shape. Then, we created MNI-aligned grids in each subjects' individual headspace. To this end, each subject's head shape was warped to the MNI template brain, and the inverse of the transformation matrix was applied onto an MNI template grid with 889 grid points and, on average, 1.5 cm spacing. With this method, we achieved a consistent mapping of the spatial positions of grid points across subjects and to the MNI template. Single-trial, MEG-sensor time courses were projected into source space (individual MNI-aligned grids) using a linear constrained minimum variance beamformer algorithm (Van Veen et al., 1997). We defined a common spatial filter based on the covariance matrix of the bandpass filtered signal (cutoff frequencies, 1–30 Hz). The covariance window depended on the locus of the effect as expected from sensor-level analysis (2–4 s). The beamformer filters were computed using the broadband single-trial data. Both the information from the magnetometer and planar gradiometer sensors systems were used. Time–frequency representations (see above) were calculated from single-trial, source-space data; its relative change to the baseline epoch (−0.5 to 0 s) was calculated (1 – signal/baseline) and then averaged between 9 and 13 Hz and 2 and 4 s. The resulting power increase maps (expressed in percent increase from baseline) were interpolated onto a standard MNI brain for illustrative purposes (see Fig. 2*C*). Anatomical structures corresponding to localized sources were found using the MNl brain and Talairach atlas (MRC Cognition and Brain Sciences Unit; imaging.mrc-cbu.cam.ac.uk/imaging/MniTalairach).

#### Statistical analysis

##### Cluster-based permutation statistics

The statistical analysis between conditions was done on the sensor-level time courses for power and bursting averaged over the gradiometer sensors with a significant power increase (Wilcoxon *z* statistic; see Fig. 2*B*) and the frequencies between 9 and 13 Hz (or 20 and 28 Hz for β-band bursting). It is important to note that this analysis strategy avoids “double dipping” because the gradiometer sensor selection as a ROI is based on the power increase from baseline across all conditions (see above). The average α-band power and burst rate over trials were compared between conditions (i.e., between tasks, between tasks at different object-pools, between different object-pools per task, and between correct vs error trials per task) across each task epoch separately (i.e., fixation epoch [0–1 s], cue epoch [1–2 s], motion epoch [2–4 s], jitter epoch [4–5 s], and delay epoch [0–1 s from delay onset]) using a nonparametric, cluster-based permutation test (Maris and Oostenveld, 2007). The β-band burst rates were tested with the same method during the cue epoch. The choice of the task epochs was based on the temporal properties of the visual stimulation. Temporal smoothing inherent in the applied sliding Wavelet-window approach can influence the observed effects close to the boundaries of the task epochs. The error bounds are within ±2.5 cycles per frequency (i.e., for 9–13 Hz: ±192–277 ms). This effect, however, dampens considerably with increasing temporal distance because of the implicit Gaussian kernel of the Wavelet window. This nonparametric, cluster-based permutation procedure controls for the Type I error accumulation arising from multiple statistical comparisons at multiple time points. First, temporal clusters of adjacent suprathreshold effects (dependent-samples *t* statistics exceeding *p* < 0.05, two-sided) were identified. The *t* values within a connected cluster were summed up as a cluster-level statistic. Then, random permutations of the data were drawn by exchanging the data between conditions within the participants. After each permutation run, the maximum cluster-level statistic was recorded, generating a reference distribution of cluster-level statistics (approximated with a Monte Carlo procedure of 1000 permutations). The proportion of values in the corresponding reference distribution that exceeded the observed cluster statistic yielded an estimated cluster-level *p* value, which is corrected for multiple comparisons.

##### Statistical analysis of neural activity increases integrated across the motion epoch

In order to statistically test the observed task- and object-pool-specific pattern for main and interaction effects, we calculated single-trial metrics of the α-band burst events (i.e., their count and duration per trial) and for α-band power across the motion and transition epochs (2–5 s). The burst counts were calculated as the number of burst occurrences per trial (see above for the burst detection algorithm) with a minimum of 1 cycle per respective frequency between them. The burst duration was calculated as the average time period over all burst events per trial (in case of multiple burst events). We only considered burst events between 2 and 5 s and thus excluded those, of which the bigger proportion was outside this time interval. Moreover, we computed the average α-band power by first subtracting the power in the baseline epoch (−0.5 to 0 s) per condition and then averaging the power values between 2 and 5 s. Power, burst counts, and durations were averaged over gradiometer sensors with a significant power increase (Wilcoxon *Z*-statistic; see Fig. 2*B*) and the frequency bins between 9 and 13 Hz, and then compared between the conditions by means of repeated-measures ANOVAS and dependent-samples *t* tests.

## Results

Behavioral performance was quantified in terms of percent correct trials, in which the difference between the responded and the probed screen location (i.e., partial report item or centroid) was <0.5 DVA. As expected, performance for both tasks decreased with more processed objects under both single- and dual-task conditions (single: *F*_(2,46)_ = 304, *p* ≤ 1 × 10^−323^; dual: *F*_(2,46)_ = 231, *p* ≤ 1 × 10^−323^; Fig. 1*C*). The main effect between the tasks (single: *F*_(1,23)_ = 19, *p* ≤ 2.3 × 10^−4^; dual: *F*_(1,23)_ = 48, *p* ≤ 4.3 × 10^−7^) is not straightforward to interpret in absolute terms (i.e., whether it is different from zero), because the two tasks differ in chance level (Alvarez and Oliva, 2008). Moreover, it depended on single- versus dual-task conditions and on the number of processed objects. As expected given their proposed difference in attention (Alvarez and Oliva, 2008, 2009), the dual-task demands impaired individuation (single vs dual: *t*_(23)_ = 7.4, *p* ≤ 1.6 × 10^−7^), while it had only negligible effects on ensemble-averaging (*t*_(23)_ = 1.1, *p* ≤ 0.28). Furthermore, individuation and averaging performance decreased differently with increasing numbers of processed objects (interaction for task × object-pool, single: *F*_(2,46)_ = 18, *p* ≤ 1.7 × 10^−6^; dual: *F*_(2,46)_ = 10, *p* ≤ 2 × 10^−4^). Consistent with different capacity limits for the two tasks, object individuation performance continued to decrease with increasing target-object pools, whereas ensemble-averaging performance remained more stable across many objects (Fig. 1*C*).

**Figure 4.**
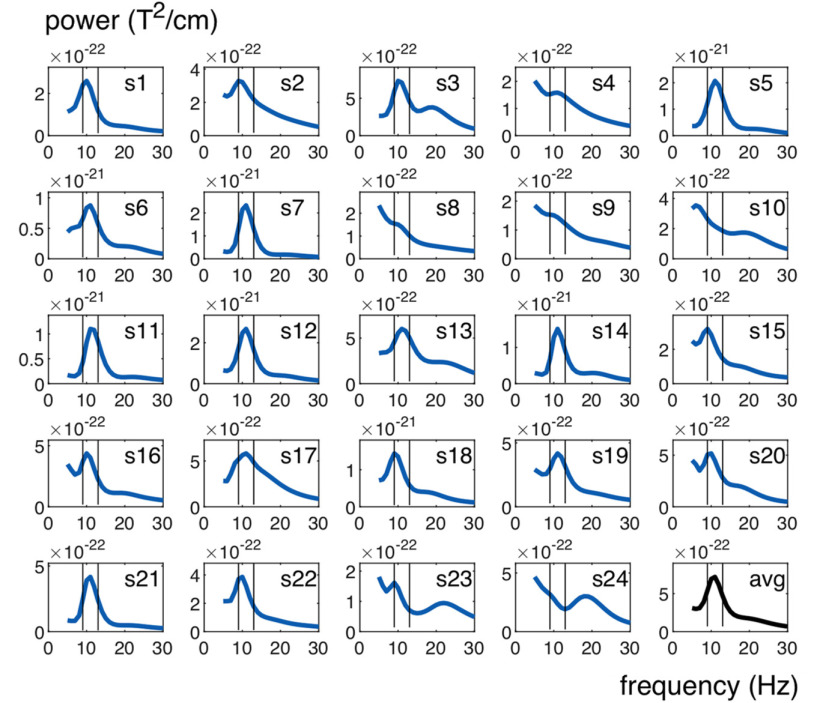
Single-subject power spectra during MOT. Power spectra averaged over significant gradiometer sensors (see Fig. 2*B*) and between 2 and 4 s for each participant separately and for the grand average. Black horizontal lines indicate the α-frequency range at 9-13 Hz.

**Figure 5.**
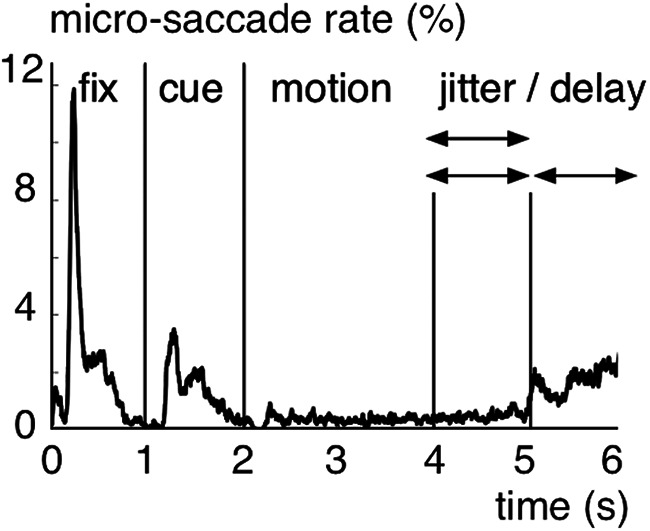
Micro-saccades during MOT. Micro-saccade rate (in percent trials).

In order to identify neural activity increases during the MOT task, we computed time–frequency power maps and used a Wilcoxon sign-rank test to compare the power increase over trials during task epochs against pretrial baseline epochs (500 ms before fixation onset; see Materials and Methods). Neural activity increased from baseline first during the target-cue epoch in the α- (9-13 Hz) and β-frequency bands (20-28 Hz). Then during the motion epoch, power remained exclusively elevated in the α-frequency band (Fig. 2*A*). The α band effects increased until ∼1 s after motion onset and ceased toward the jittered transition from the motion into the delay epoch. In separate analyses, we time-locked the power maps to the end of the motion epoch on each trial to test for exclusive delay epoch activity. It showed that α-band power was still significantly increased during the memory delay (Fig. 2*A*). The α-band power increase had a central-parietal topography (8 significant MEG-gradiometer channels; *p* < 0.05, one-sided and Bonferroni corrected; Fig. 2*B*). We used an lcmv-beamforming algorithm (Van Veen et al., 1997) (see Materials and Methods) to locate the neural generators of the observed α-band effects. In line with prior fMRI work on individuation for static displays (Todd and Marois, 2004; Xu and Chun, 2009), we found the greatest signal change during the motion epoch versus pretrial baseline epochs in the inferior parietal cortex (BA 40; Fig. 2*C*). The source modeling revealed bilateral cortical activations, but the strongest effects were found in the left hemisphere (maximum voxel MNI coordinates in cm = [−50, −50, 55]). Similar results were found on MEG-magnetometer channels (10 significant sensors; Fig. 3*A*) and when using a random-effects approach to quantify the α power increase from baseline (dependent-samples *t* test for BL vs motion epoch over participants; Fig. 3*B*). On a single-subject-level, 22 of 24 participants showed a peak between 9 and 13 Hz during the motion epoch (average peak at mean = 10.5 Hz, SD = ±2 Hz; Fig. 4). We recorded the participants' eye gaze to control for micro-saccades during MOT. The long motion epoch with multiple moving objects favors micro-saccades, which can mirror oscillatory neural activity (Yuval-Greenberg et al., 2008) and alter perception and attentional selection (Hafed, 2013). In our paradigm, however, effects resulting from micro-saccades were negligible because they occurred rarely during the motion epoch (0.3%–0.5% trials; Fig. 5). Further, there were no significant correlations between the micro-saccade size and the simultaneously recorded α power across micro-saccade occurrences for none of the participants (false discovery rate-corrected for multiple-comparison across participants, all absolute *r* < 0.39, all false discovery rate-adjusted *p* > 0.48) (Benjamini and Hochberg, 1995).

**Figure 6.**
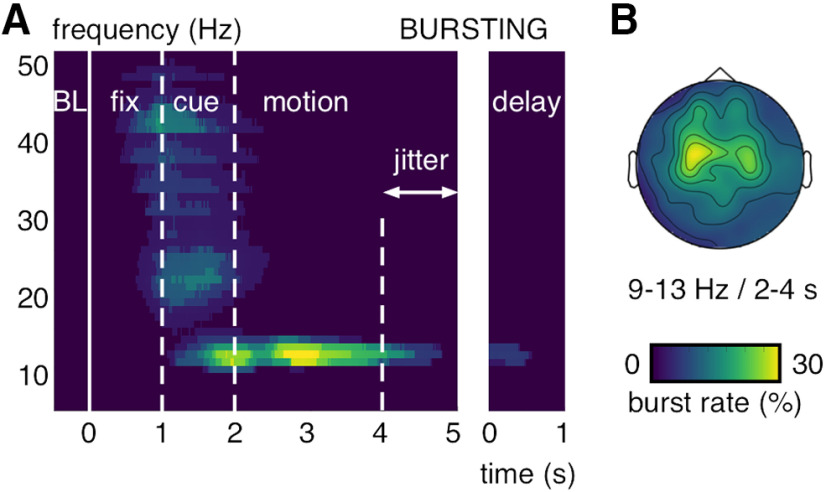
α-band bursting during MOT. ***A***, Burst rate (in percent trials) averaged over significant gradiometer sensors (see Fig. 2*B*). Burst rates >25% are shown. ***B***, MEG-gradiometer topography for the burst rates (in percent trials) averaged between 9 and 13 Hz and 2 and 4 s.

**Figure 7.**
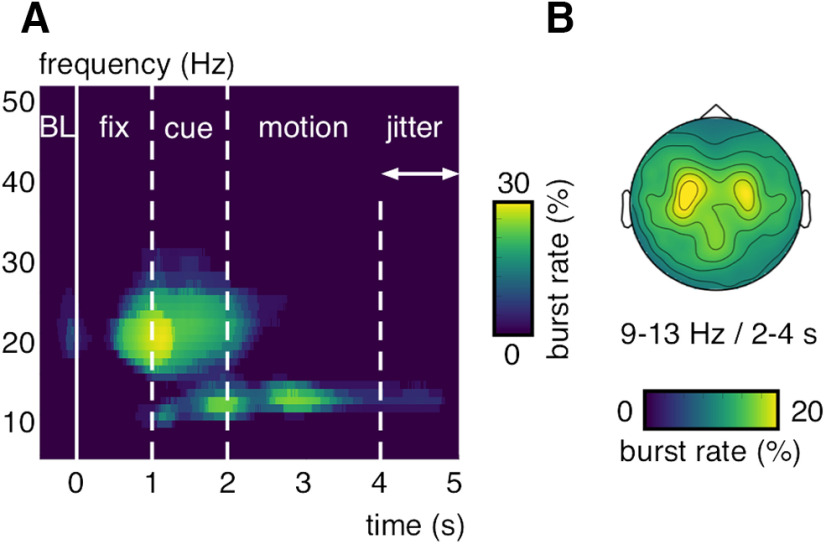
α-band bursting during MOT with an alternative algorithm. ***A***, Burst rate (in percent trials) computed with an alternative algorithm (Quiroga et al., 2004) averaged over significant gradiometer sensors (see Fig. 2*B*). Burst rates >20% are shown. ***B***, MEG-gradiometer topography for the burst rates (in percent trials) averaged between 9 and 13 Hz and 2 and 4 s.

We used bursting analysis methods (Lundqvist et al., 2016) (see Materials and Methods) to compute the observed power increases on the single-trial level and compare it between the different experimental conditions. In contrast to standard across-trials averaged power analyses, the bursting approach isolates time- and band-limited, high-signal epochs (i.e., bursts) during each trial with respect to its respective pretrial mean and variability estimate. Because bursts can occur at different rates, times, and with different durations from trial to trial, we expected them to be more diagnostic of dynamic activity changes in the time-critical MOT task. We found the strongest bursting activity, quantified in the percentage of trials that contained a burst at a particular sensor-time–frequency sample (i.e., burst rate), in the α-frequency band during the motion epoch over central-parietal MEG-gradiometer sensors (Fig. 6*A*,*B*). Similar results were found on MEG-magnetometer channels (Fig. 3*C*), without subtracting event-related fields from single trials (Fig. 3*D*) and with an alternative algorithm for burst detection (Quiroga et al., 2004) (see Materials and Methods; Fig. 7). On average, one or two α-band bursts were detected per trial (mean = 1.15; SD = 0.22) with a mean duration of 865 ms (SD = 348 ms; see Fig. 10).

**Figure 8.**
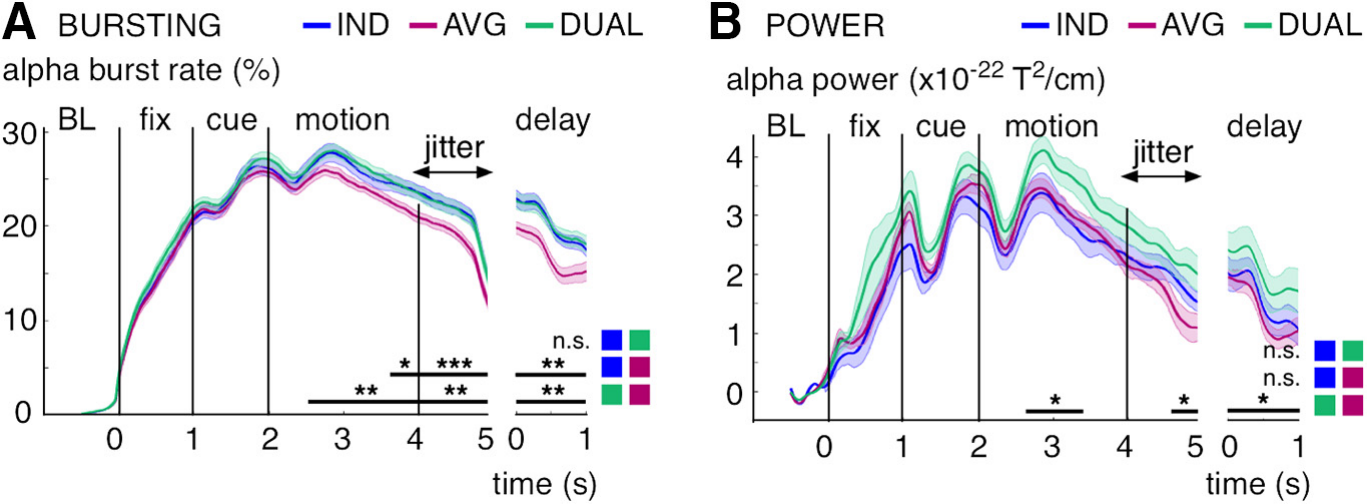
α-band bursting and power increase separated by experimental task. ***A***, Burst rate (in percent trials) per task averaged between 9 and 13 Hz and over significant gradiometer sensors (see Fig. 2*B*). ***B***, Power per task averaged between 9 and 13 Hz and over significant gradiometer sensors (see Fig. 2*B*). Shaded regions represent 1 SE for repeated measures (Morey, 2008). Black lines indicate significant effects (cluster-corrected). *p < 0.05, **p < 0.01, ***p < 0.005, n.s., not significant.

The single-trial bursting analysis was sensitive to differences in neural activity between the performed tasks during MOT (individuation, averaging, dual-task). We observed greater α-band burst rates for individuation versus averaging during the motion epoch (cluster-corrected *p* ≤ 0.05), during the jittered transition between motion and delay epochs (cluster-corrected *p* ≤ 0.004), and during the delay epoch (cluster-corrected *p* ≤ 0.006; Fig. 8*A*). Similar results were found for the dual-task condition versus averaging (motion: all cluster-corrected *p* ≤ 0.008, jitter: *p* ≤ 0.006, delay: *p* ≤ 0.006). By contrast, we found nosignificant differences for the dual-task condition versus individuation during any task epoch. Moreover, there were no significant differences in the α-band burst rates during the fixation and cue epochs and in β-band bursting (averaged between 20 and 28 Hz) during the cue epoch between any of the tasks. The observed α band effects rooted mainly on single-trial activity changes because across-trials averaged power was less sensitive to the between-task differences (Fig. 8*B*). We used baseline-corrected power (baseline interval: −0.5 to 0 s), which factors out average baseline differences between the conditions, but in contrast to the burst analysis, it does not quantify activity increases from baseline for each single trial. We found no α-band power differences for individuation versus averaging and for individuation versus the dual-task condition. However, there was greater α-band power for the dual-task versus averaging (motion: all cluster-corrected *p* ≤ 0.022, jitter: *p* ≤ 0.024, delay: *p* ≤ 0.034).

**Figure 9.**
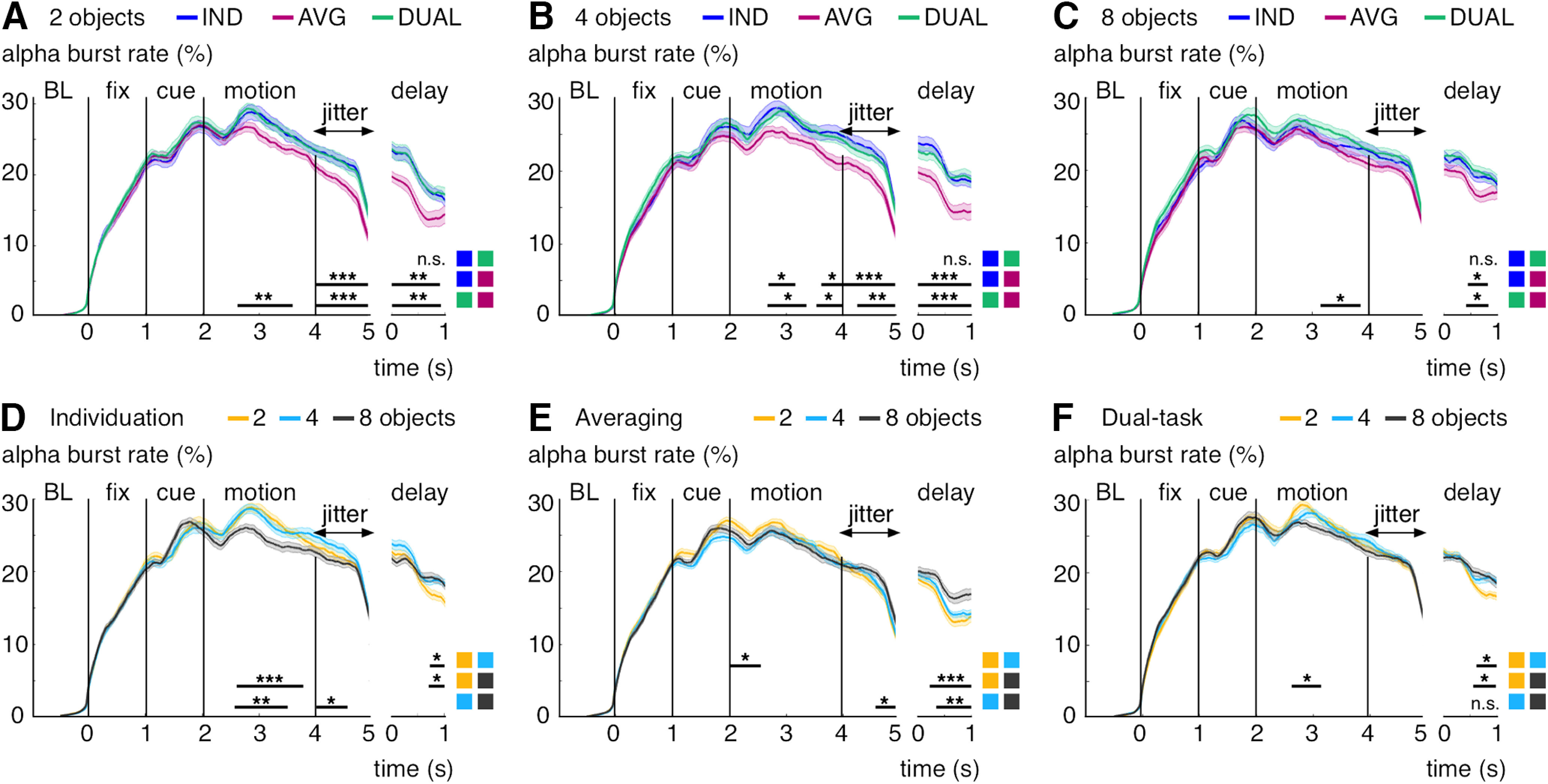
α-band bursting during MOT separated by task and object-pools. ***A-C***, Burst rate (in percent trials) per task averaged between 9 and 13 Hz and over significant gradiometer sensors (see Fig. 2*B*) for two (***A***), four (***B***), and 8 objects (***C***). ***D-F***, Burst rate (in percent trials) per object-pool averaged between 9 and 13 Hz and over significant gradiometer sensors (see Fig. 2*B*) for individuation- (***A***), averaging- (***B***), and dual-task conditions (***C***). Shaded regions represent 1 SE for repeated measures (Morey, 2008). Black lines indicate significant effects (cluster-corrected). *p < 0.05, **p < 0.01, ***p < 0.005, n.s., not significant.

The observed α-band bursting effects were strongest up to the typical capacity limitations for multiple-object processing at ∼4 objects. We found significant differences between the individuation versus averaging tasks below/at capacity during the motion epoch (4 objects: cluster-corrected *p* ≤ 0.038 and *p* ≤ 0.04), during the motion-delay transition (2 objects: all cluster-corrected *p* ≤ 0.002, 4 objects: *p* ≤ 0.004), and during the delay epoch (2 objects: all cluster-corrected *p* ≤ 0.006, 4 objects: *p* ≤ 0.002). Above capacity at 8 objects, however, there were only small differences between the two tasks during the delay (cluster-corrected *p* ≤ 0.044; Fig. 9*A–C*). The dual-task condition showed no significant differences versus individuation for any task epoch or object pool. However, it showed greater burst rates versus averaging below/at capacity (2 objects, motion: all cluster-corrected *p* ≤ 0.01, jitter: *p* ≤ 0.004, delay: *p* ≤ 0.008; 4 objects, motion: *p* ≤ 0.022 and *p* ≤ 0.042, jitter: *p* ≤ 0.01, delay: *p* ≤ 0.004) and smaller effects above capacity (8 objects, motion: all cluster-corrected *p* ≤ 0.02, delay: *p* ≤ 0.034; Fig. 9*A–C*).

Comparing the different object-pools for each task condition separately showed that capacity limitations had a strong impact on the burst rates during the motion epochs for individuation and, in part, under dual-task conditions. We found greater burst rates below versus above capacity (individuation 2 vs 8 objects, motion: all cluster-corrected *p* ≤ 0.002; 4 vs 8 objects, motion: *p* ≤ 0.008; 4 vs 8 objects, jitter: *p* ≤ 0.018; dual task 2 vs 8 objects, motion: *p* ≤ 0.02) but no significant differences below/at capacity (i.e., 2 vs 4 objects; Fig. 9*D*,*F*). By contrast, we found a different pattern on averaging trials. There were burst rate differences below/at capacity (2 vs 4 objects, motion: cluster-corrected *p* ≤ 0.022) and more bursting above versus below capacity (4 vs 8 objects, jitter: cluster-corrected *p* ≤ 0.022; Fig. 9*E*). Moreover, the burst rates during the delay epoch were also different for individuation/dual-task conditions versus averaging. Whereas we found particularly low burst rates on two-object trials under individuation (2 vs 4 objects: all cluster-corrected *p* ≤ 0.036; 2 vs 8 objects: *p* ≤ 0.044) and dual-task conditions (2 vs 4 objects: all cluster-corrected *p* ≤ 0.026; 2 vs 8 objects: *p* ≤ 0.012), averaging was mainly characterized by stronger burst rates on 8 object trials (2 vs 8 objects: all cluster-corrected *p* ≤ 0.004; 4 vs 8 objects: *p* ≤ 0.006; Fig. 9*D–F*).

**Figure 10.**
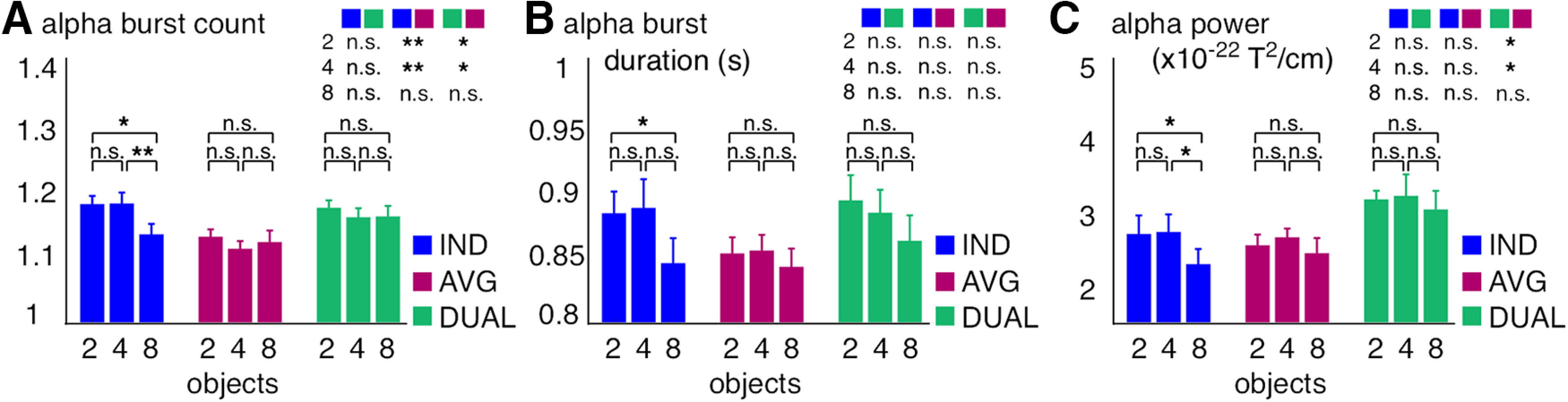
α-band burst count, burst duration, and power during MOT. ***A-C***, Burst count per trial (***A***), burst duration per trial (***B***), and power (***C***) for each task and at different object-pools averaged between 9 and 13 Hz and between 2 and 5 s and over significant sensors (see topography in Fig. 2*B*). Error bars indicate 1 SE for repeated measures (Morey, 2008). Significance level for dependent-samples *t* tests between each condition: *p < 0.05, **p < 0.01, n.s., not significant.

In order to formally test the observed task- and object-pool specific pattern, we ran two-way, repeated-measures ANOVA and *post hoc*, dependent-samples *t* tests on the number of α-band bursts per trial, their average duration, and the average α-band power (detected and integrated from 2 to 5 s after motion onset; Fig. 10). For the α-band burst count per trial, we found a significant main effect for the individuation versus averaging tasks (*F*_(1,23)_ = 5.9, *p* ≤ 0.023) and an interaction between the performed tasks across different numbers of processed objects (*F*_(2,46)_ = 3.9, *p* ≤ 0.028). There were more bursts on individuation versus averaging trials for two (*t*_(23)_ = 3, *p* ≤ 0.007) and 4 objects (*t*_(23)_ = 3, *p* ≤ 0.007) but not for 8 objects (*t*_(23)_ = 0.5, *p* > 0.62). By contrast, there were no significant main and interaction effects for the α-band burst duration and power. Comparing individuation versus the dual-task condition, we found a significant interaction over object-pools for the burst counts (*F*_(2,46)_ = 3.9, *p* ≤ 0.028) and no significant effects for the burst duration and power. Moreover, for averaging versus the dual-task condition, there were main effects on the burst counts (*F*_(1,23)_ = 6.9, *p* ≤ 0.016) and on power (*F*_(1,23)_ = 5.6, *p* ≤ 0.027) but no significant effects for the burst duration (Fig. 10).

**Figure 11.**
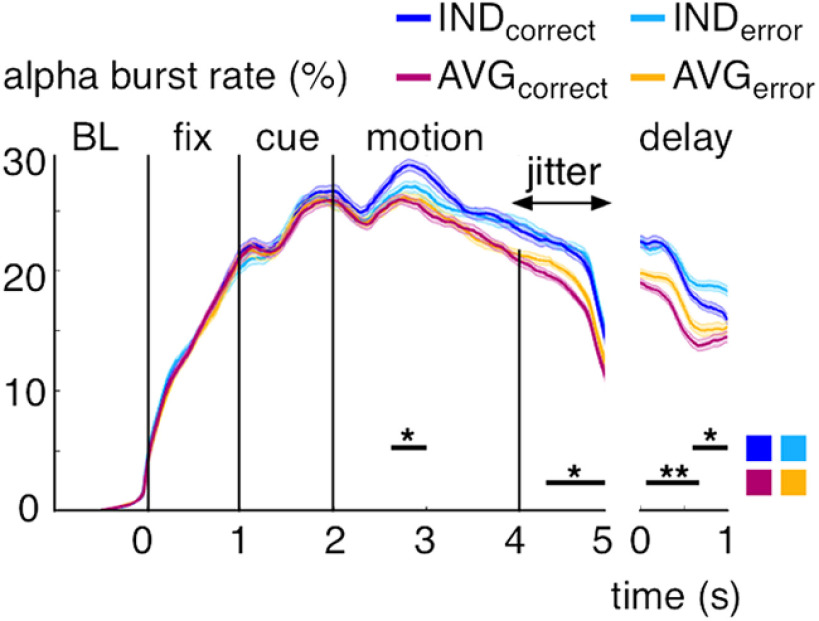
α-band bursting during MOT separated by correct versus error trials. Burst rate (in percent trials) per task averaged between 9 and 13 Hz and over significant gradiometer sensors (see Fig. 2*B*) separately for correct and error trials. Shaded regions represent 1 SE for repeated measures (Morey, 2008). Black lines indicate significant effects (cluster-corrected). *p < 0.05, **p < 0.01.

Finally, we investigated possible links between bursting and behavioral performance, to add support for the behavioral relevance of our results. To this end, we split correct and error trials separately for individuation and averaging conditions and contrasted their α-band burst rates over each task epoch. For individuation, we found greater burst rates on correct trials during the motion epoch (cluster-corrected *p* ≤ 0.05) and greater burst rates on error trials during the delay epoch (cluster-corrected *p* ≤ 0.02; Fig. 11). For averaging, there were greater burst rates on error trials during both the jitter epoch (cluster-corrected *p* ≤ 0.018) and during the delay epoch (cluster-corrected *p* ≤ 0.008). There were no significant differences during the other task epochs (Fig. 11). These results suggest that the observed bursting effects were relevant for behavior in different ways for the two tasks.

## Discussion

We observed oscillatory bursts over inferior parietal cortex during dynamic multiple-object situations. Confirming previous work on dynamic multiple-object processing, we observed effects in the α-frequency band (Rouhinen et al., 2013; Jia et al., 2017). Advancing on previous reports, however, the burst occurrences were diagnostic for the task demands for object individuation versus ensemble/average processing, the number of processed objects during MOT, and its behavioral outcome. On individuation trials, more bursts were detected below/at versus above the typical capacity limit. By contrast on ensemble trials, we found more stable burst rates across different object-pools, which were lower compared with those found on individuation trials up to capacity. The observed bursting effects occurred both “online” during object-motion tracking and “offline” during the memory delay. Moreover, under dual-task conditions, we found greater burst rates compared with single-task ensemble averaging and similar burst rates compared with single-task individuation, which suggests strategic influences per task on the observed effects. Finally, the observed bursting showed behavioral relevance because greater bursting was found on correct individuation trials and on error trials for averaging. Overall, our findings identify the neural correlates for object versus ensemble and below versus above capacity processing during time-critical, multiple-object scenarios.

α-band oscillations are thought to regulate neural excitability via inhibitory control (Klimesch et al., 2007; Haegens et al., 2011). Along these lines, two possible functional interpretations for the observed dynamic α band increases stand out: On the one hand, they could reflect the inhibition of task-irrelevant inputs (Bonnefond and Jensen, 2012; Roux and Uhlhaas, 2014). Previous work on event-related potentials has suggested that both effects resulting from target enhancement and distractor suppression play a role during MOT (Drew et al., 2009; Doran and Hoffman, 2010). Less target-objects meant also more distractor-objects, respectively, in our paradigm. Thus, in line with an interpretation based on distractor suppression, we found greater α-band burst rates with greater numbers of task-irrelevant objects. Alternatively, the observed α band effects might signal inhibitory control needed to prioritize, select, and maintain task-relevant objects carved out as individuals from visual inputs (Palva and Palva, 2007; Jensen et al., 2014; Wutz et al., 2014). In this perspective, complex visual scenes are segmented into local objects by regulating neural excitability focally according to their α-timed release from inhibition. By contrast, inhibition is more globally distributed across many objects in the scene for ensemble computations. Consistent with this view, we found greater α-band burst rates up to the typical capacity limits for object individuation and similar burst rates below capacity. Above capacity, however, when individuation via α-timed inhibitory control is insufficient, we observed lower α-band bursting down to the level for global ensemble processing. The observed effects during the delay epoch further support the interpretation based on task-relevant object individuation. No task-irrelevant distractors were present during the memory delay; thus, effects resulting from distractor suppression were not expected. Instead, during the delay, task-relevant objects need to be kept separate via inhibitory control to perform partial report on individuation trials. By contrast, on ensemble trials, the centroid task allowed for integration and maintenance in a compressed format (i.e., their average) without explicitly representing the spatial positions of the individual objects during the delay (Greene and Oliva, 2009; Alvarez, 2011; Corbett and Oriet, 2011). Importantly, we also found α-band bursting differences between the two tasks during the motion epoch. In addition to its effects on memory maintenance, this suggests that α-timed inhibitory control can also be used dynamically to switch between input individuation and compression “online” during dynamic multiple-object perception.

Alternatively, the observed α-band bursting effects reflect rather differences between the two tasks in terms of difficulty or effort. Three lines of evidence render this explanation inadequate in our paradigm. First, we found the greatest burst rate differences when performance was most similar (below/at capacity) and the smallest burst rate differences when the performance difference was greatest between the two tasks (above capacity, see Behavioral data; Fig. 1*C*). Second, behavioral outcome impacted bursting differently for the two tasks with more bursting on correct individuation trials and on incorrect averaging trials. Third, whereas the dual-task demands worsened individuation performance probably because of higher difficulty, it did not impact the burst rates under dual-task conditions versus single-task individuation. Instead, we found burst rate differences between the dual-task condition versus single-task averaging. This pattern suggests that the observed effects were because of top-down strategies during single-task ensemble-averaging, which could not be applied under single-task individuation and dual-task conditions. The investigation of top-down grouping strategies during multiple-object processing has a long history in experimental and cognitive psychology (Wertheimer, 1912; Yantis, 1992; Merkel et al., 2014, 2015, 2017). The observed α-band bursting effects signal a potential implementation of top-down principles during dynamic multiple-object scenarios into neural function by grouping objects into ensemble representations.

Our findings queue up with recent reports from neurophysiology about oscillatory burst events underlying cognitive and motor operations (Feingold et al., 2015; Lundqvist et al., 2016; Sherman et al., 2016; Shin et al., 2017). These observations have sparked a debate on principles about the sustained versus transient nature of oscillatory dynamics (for discussion, see van Ede et al., 2018). The key aspect is that, in many cases, sustained oscillatory activity represents an analysis artifact from averaging over trials, and it may instead be better captured by transient high-signal burst events that happen at different rates and times, and with different durations from trial to trial. Here, we detected bursts in single-trial MEG activity. It is thus not clear whether and how our findings relate to the oscillatory burst events found with single-cell neurophysiology. Critically, however, the bursting analysis served as a sensitive tool to capture single-trial differences in our paradigm, which would have gone unnoticed with a standard approach on across-trials averaged power. Comparing the dynamic mode of attention to multiple moving objects studied here versus classic “sustained attention” situations, in which typically more sustained power effects are found, buttresses the potential functional significance of the observed bursting effects. The following three aspects stand out: First, in a classic “sustained-attention” task, participants typically need to constantly monitor a static stimulus at a particular fixed spatial location. By contrast, our MOT task requires keeping track of dynamic visual objects, which continuously change their spatiotemporal positions in random directions throughout the trial. Second in contrast to “sustained-attention” over time to a single, isolated object or spatial location, the participants need to dynamically shift their attention-focus multiple times per trial between many objects/locations during MOT. Third, a typical attention experiment is built on a repetitive trial structure that reiterates the same sequence of temporal events often with the same constant latencies over and over (e.g., Cue – Focus – Response). In our MOT task, however, not one single trial is like the next one because the spatiotemporal coordinates of each object's motion path were randomly generated for each trial. Consequently, there is a high degree of intertrial variability in the timing of the relevant perceptual/cognitive processes, because critical situations might happen at different times on different trials (e.g., when an object moves to the display edges or 2 objects cross in close proximity).

All three aspects (static vs motion, one vs many objects, intertrial variability) favor sparse bursts over sustained activity. Moreover, they suggest that MOT requires a more dynamic mode of attention compared with the typical “sustained attention” situation. Importantly, these three aspects are also more pronounced on individuation versus averaging trials. For individual objects, there is (1) “more” motion (i.e., a higher dynamic range in the spatiotemporal coordinates vs a more stable centroid), (2) “more” objects (i.e., processing is split between multiple objects vs one average object), and (3) “more” intertrial variability (i.e., in timing for independent objects vs averages). Because MOT for individual objects versus ensembles requires more dynamic and flexible attention, it is better captured by sparsely timed bursts on individual trials compared with across-trials averaged, sustained activity.

This view is in line with recent perspectives from neurophysiology and computational modeling on dynamic processing and its burst-like neural signatures. For example, in the Working Memory 2.0 model (Miller et al., 2018), brief bursts of oscillatory activity reflect reactivations of attractor states for new mental content (i.e., new attention foci or working memory items), beyond the established view based on sustained and persistent activity. Neural ensemble communication in α and β oscillations (there is explicitly no distinction between the two in the theory) acts as a default state and implements top-down information and inhibition. Excitation at sensory read-in kicks the rhythms up to γ frequencies in short discrete bursts supporting the encoding of new content and refreshing previous impressions. α/β- and γ-frequency bursts serve antagonistic roles on inhibition-excitation. Its interplay enables the exertion of the volitional, executive control needed to act flexibly in dynamic situations. α/β inhibition is integral in the model because it allows for selective and timed routing of sensory flow into objects. Consequently, more α/β bursting would be expected for many individual objects versus one average ensemble. In principle, attractor networks are capable of processing multiple items simultaneously with minimal interference between them through temporal multiplexing their reactivations in time. However, the refresh rate of successive reactivations sets the system's capacity limitations. Consistent with our observations, exceeding capacity would then lead to a breakdown of bursting because too many items have to be reactivated within the limited time available for the refresh.

Our results constitute an important step toward understanding how dynamic multiple-object processing is implemented in brain function. Consistent with our source modeling results, most functional explanations for object and scene processing thus far revolve around spatially defined saliency maps in parietal cortex (Todd and Marois, 2004; Xu and Chun, 2009). Our findings augment these theoretical perspectives by capturing its temporal dynamics in neural α band oscillations. They may thus lead the way to novel conceptions about how dynamic multiple-object situations are solved in real time in the visual world and in computer vision (supporting recent developments in computational modeling and engineering) (Mahadevan and Vasconcelos, 2010; Nguyen et al., 2013). Alpha oscillations are thought to coordinate the timing of brain processes (Klimesch et al., 2007; Klimesch, 2012) and to underlie the temporal resolution of visual perception and attention (Gho and Varela, 1988; VanRullen et al., 2005; Samaha and Postle, 2015; Wutz et al., 2018). They structure visual processing into discrete temporal events (for review, see VanRullen and Koch, 2003), within which scenes are decomposed into objects via the timed release from inhibition (for review, see Jensen et al., 2014; Wutz and Melcher, 2014). Our findings reveal that this spatiotemporal event structure can be dynamically regulated between object- and ensemble-analysis levels, to overcome capacity limitations because of selective attention and to master the real-time requirements of visual cognition.
